# Polypropylene Pipe Compounds with Varying Post-Consumer Packaging Recyclate Content

**DOI:** 10.3390/polym14235232

**Published:** 2022-12-01

**Authors:** Paul J. Freudenthaler, Joerg Fischer, Yi Liu, Reinhold W. Lang

**Affiliations:** 1 Institute of Polymeric Materials and Testing, Johannes Kepler University Linz, Altenberger Straße 69, 4040 Linz, Austria; 2 Borealis Polyolefine GmbH, Innovation Headquarters, St. Peterstraße 25, 4021 Linz, Austria

**Keywords:** plastics recycling, pipe materials, fatigue crack growth, cracked round bar, polypropylene, post-consumer, recyclate

## Abstract

The high recycling targets set by the European Commission will create an increased availability of polypropylene (PP) post-consumer recyclates (PCRs). However, no regulations mandate the use of recycled PP (rPP), so the industry is challenged to explore possibilities to utilize such materials. One option, as suggested by the European Commission, is the introduction of rPP in pipe applications. According to existing standards, the use of recyclate is not allowed in pressurized gas and drinking water systems. However, many other pipe and underground applications, such as stormwater systems, open the increased use of PCRs. Additionally, even for less-demanding applications, such as non-pressure sewage systems, highly durable solutions are needed to cover the requested lifetime and request an ambitious property profile to fulfill the application needs that cannot be met by PP packaging materials and even less by PCRs thereof. Hence, this work explores the possible use of commercially available PCRs out of polypropylene from packaging applications in compounds together with virgin PP pipe grades to meet the demands for less-demanding applications. Two different commercially available rPPs and one commercially available recycled polyolefin (rPO) from mixed polyethylene and PP waste were acquired and, together with two predefined virgin PP pipe grades, were blended to compounds in the range of 10 m%, 20 m%, and 30 m% recyclate content. The compounds and three virgin PP pipe grades, acting as benchmarks, were tested in terms of short- and long-term mechanical performance as well as for many other physical properties. All of the compounds showed good results regarding fatigue crack (FCG) resistance with virgin polymer as the reference. The factors influencing FCG resistance, such as melt flow rate and polyolefin cross-contamination, were thoroughly investigated as the used virgin grades and recyclates cover a broad range of these properties.

## 1. Introduction

Within the European Union, the European Commission pushes for the development of a more circular plastics economy by declaring goals for the use of recycled plastics in new products [1] and recycling targets for plastic packaging waste [2]. While regulations exist for the use of recycled polyethylene terephthalate (rPET) within beverage bottles [3], there is no regulation mandating the use of recycled polypropylene (rPP) in any application. Since there is a recycling target for PP waste but no mandatory use of rPP in any application, the industry is challenged to explore suitable applications for the utilization of these post-consumer recyclates (PCRs). One such application, according to the European Commission [1], is the use of PCRs in non-packaging applications such as pipes, as they show “good potential for uptake of recycled content”.

The different European pipe standards for PP sewage pipes, DIN EN 1451-1 [4], DIN EN 1852-1 [5], and DIN EN 13476-2 [6] and -3 [7], at the time this paper is written, strictly forbid the use of “material from PP products other than pipes and fittings” in PP pipes. While this is completely understandable for highly demanding applications such as pressurized drinking water pipes and pressurized gas pipes, rPP from “PP products other than pipes and fittings” might still be considered to partly replace virgin materials in non-pressure less-demanding piping applications. Suitable applications would be, e.g., drainage, sewerage, or irrigation, if the material fulfills the required property profile.

Apart from the defined origin of the material, all of the cited standards have the same three material characteristic demands of the pipe resin: a melt flow rate [8] below a certain limit, a suitable thermal stability via an oxidation induction time [9] over 8 min, and resistance to internal pressure [10] for a certain amount of time at specified temperatures and pressures. Apart from these parameters for the pipe resin, the produced pipes have to pass a myriad of component-level tests, e.g., ring stiffness [11], impact strength tested on a pipe [12], the ring flexibility [13], etc. While many of these parameters can at least be predicted by specimen-level tests, e.g., tensile tests [14], Charpy notched impact tests [15], and fatigue crack growth experiments [16], etc., these specimen-level tests can only act as pretests and can be used for the development of applicable compounds. However, they do not compensate for component-level tests.

There are previous experiments showing the successful incorporation of rPE from different sources with virgin PE100 and PE100-RC pipe grades [17,18]. Many of the produced compounds fulfill several short- and long-term performance parameters required for use within less-demanding pipe applications. No such preceding work was found for PP. The overall objective of the present work is to show the applicability of PP PCRs in pipe compounds for less-demanding PP pipe applications and to contribute to future developments in this field as the first step toward pipe products.

## 2. Materials

Three different recyclate grades were provided by two European recycling companies, two of them designated as “rPP” for recycled polypropylene (PP) from post-consumer PP packaging waste, and one of them designated as “rPO” for recycled polyolefin from mixed post-consumer polyethylene (PE) and PP waste. All three recyclates were delivered as pellets and will henceforth be called rPP-A, rPP-B, and rPO-C. While the main feedstock for the recyclates was the “yellow-bag” system, which is a separate collection stream for plastic packaging products in Germany and Austria [19], the recyclates passed different sorting, washing, and recycling steps and differ in color (rPP-A grey, rPP-B white, and rPO-C anthracite) and properties.

Virgin PP block-copolymer drainage pipe-grade materials were acquired for comparison and compounding in the form of pellets. The two grades, which will henceforth be called PP-1 and PP-2, are extrusion-grade materials with a specified melt flow rate (MFR) of 0.3 g/10 min at 230 °C and 2.16 kg load. These two PP pipe grades represent the upper-performance benchmarks and are used for the compounding of the recyclate pipe compounds. The third grade, PP-3, with its advertised MFR of 0.8 g/10 min, is intended to be used, e.g., for the injection molding of pipe fittings, and represents the lower performance benchmark.

All of the recyclate pipe compounds were produced on a Leistritz ZSE MAXX 18 twin-screw extruder (Leistritz Extrusionstechnik GmbH, Nuremberg, Germany) with an L/D ratio of 40D, co-rotating screws, a screw speed of 400 rpm, and a mass throughput of around 8–10 kg/h. Three gravimetric feeders, two Brabender DSR28 for the pellets and one Brabender Minitwin for stabilizer powder (Brabender Technologie GmbH & Co. KG, Duisburg, Germany) were used to ensure a consistent ratio of the virgin material, the recyclate, and the stabilization mixture to ensure no further degradation occurs during the compounding of the materials. The stabilization for each compound consisted of 0.15 m% Irganox^®^ 1010 (BASF SE, Ludwigshafen am Rhein, Germany), a phenolic primary antioxidant for long-term thermal stabilization [20], and 0.15 m% Irgafos^®^ 168 (BASF SE), a hydrolytically stable phosphite secondary antioxidant for processing stabilization [21]. The stabilization of the materials should not influence the mechanical properties measured within the scope of this paper, as no aging was applied to the specimens, and no lengthy tests in media and/or at elevated temperatures were conducted. Nevertheless, the effect of the applied stabilization on the resistance to thermal oxidation was investigated in the present paper.

Pretests of the benchmark materials concluded that PP-1 demonstrates the highest slow crack growth (SCG) resistance. Therefore, compounds containing PP-1 are expected to deliver the highest possible performance. Blends containing PP-1 and 10 m%, 20 m%, and 30 m% recyclate were produced with rPP-A and rPP-B, respectively. The third recyclate, rPO-C, was only produced with a concentration of 20 m% for comparison reasons. Additionally, rPP-A was also compounded with PP-2 in concentrations of 10 m%, 20 m%, and 30 m% recyclate to investigate the influence of the used virgin grade. Compounds containing PP-1 and rPP-A are henceforth called 1A10, 1A20, and 1A30, those containing PP-1 and rPP-B are called 1B10, 1B20, and 1B30, the compound containing PP-1 and 20 m% rPO-C will be called 1C20, and the compounds containing PP-2 and rPP-A will be called 2A10, 2A20, and 2A30. A list of all compounds together with the blending ratio is presented in Table 1.

## 3. Methods

### 3.1. Melt Flow Rate Measurements

The melt flow rate (MFR) measurements were conducted at 230 °C under a 2.16 kg static load on a Zwick/Roell Mflow melt flow indexer (ZwickRoell GmbH & Co. KG, Ulm, Germany) according to ISO 1133-1 [8] and ISO 19069-2 [22]. The cuts were made every 3 mm piston movement. The time between the cuts was measured, and each extrudate was weighed on an ABS 220-4 electronic balance (Kern & Sohn GmbH, Balingen-Frommern, Germany). The extra- and interpolation to 10 min calculated the MFR in g/10 min for each cut. For each material, one measurement was conducted. Within one measurement, 6 cuts were made and used for the calculation of average values and standard deviations.

### 3.2. Specimen Production

According to ISO 19069-2 [22], the tensile and impact test specimens for PP should always be injection-molded. Therefore, all of the multipurpose specimens (MPS) were produced via injection molding according to ISO 3167 [23] on an Engel Victory 60 (Engel Austria GmbH, Schwertberg, Austria) with a 25 mm cylinder. As the MFR test results in the next chapter will show that all compounds fall within the MFR range of <1.5 g/10 min, for injection molding, a melt temperature of 255 °C was chosen as prescribed by ISO 19069-2. All of the specimens were conditioned at 23 °C and 50% relative humidity for 3–5 days. After conditioning, these specimens were used for tensile testing, and after subsequent cutting to Type 1 specimens and notching, both in accordance with ISO 179-1 [15], they were also used for Charpy notched impact testing.

Cracked round bar (CRB) specimens for fatigue crack growth experiments were produced according to the testing standard ISO 18489 [16]. As this standard only states the processing parameters for PE, the compression molding temperature for the production of plates was elected by following the compression molding guidelines of ISO 19069-2 [22]. For specimen production, plates of the size 16 mm × 120 mm × 150 mm were compression-molded in a specifically designed positive mold with the help of a hydraulic press from the Langzauner Perfect line (Langzauner GmbH, Lambrechten, Austria). Within the fully automated program, 270 g pellets were heated within the mold from room temperature to 210 °C with the weight of the mold on top of the material. An integrated temperature sensor allows for the direct measurement of the mold temperature, and when the internal temperature of 210 °C was reached, it was held for 15 min. After that, slow cooling with a cooling rate of 2 K/min was started. The full pressure of 10 MPa was applied to the material upon reaching around 165 °C. Applying full pressure at higher temperatures leads to too much melt displacement. After reaching 40 °C, the pressure was released, the mold opened, and the plate was manually removed. The produced plates were conditioned at 23 °C and 50% relative humidity for at least three days before being cut into bars and lathed on an EMCO turning lathe (EMCO GmbH, Hallein, Austria) to CRB specimens according to ISO 18489. Therefore, round specimens with a diameter of 14 mm and a circumferential notch with a depth of 1.5 mm (resulting in a ligament diameter of 11 mm) and M14 × 1.25 threads on both sides for clamping were produced. A 0.3-mm-thick general-purpose razor blade was used to notch the specimen. Before testing, the specimens were conditioned at 23 °C and 50% relative humidity for another day after being notched.

### 3.3. Density Measurements

The density measurements were conducted according to ISO 1183-1 [24] with a Sartorius CPA 225D lab balance (Sartorius AG, Göttingen, Germany). The samples were cut from the sprue-sided shoulders of MPS. In the first step, the respective sample was weighed dry, measuring its mass in air (m_S·A_). In the second step, the sample was immersed in deionized water and put below a buoyancy cage connected to the scale, enabling the measurement of the sample buoyancy (m_S·IL_) without the need for a sinker. A wire was used to free the sample of air bubbles, and the temperature of the immersion liquid was recorded for the calculation of its density (ρ_IL_). The sample density was calculated according to Formula (1) with measurement apparatus correction variables A and B:

(1) $$\rho_{s}=\frac{m_{S\cdot A}{\;*\;\rho }_{\mathrm{IL}}}{A\;*\;\left(m_{S\cdot A}-m_{S\cdot \mathrm{IL}}\right)}+B$$

For each material, five samples, each cut from an individual MPS, were used for the calculation of average values and standard deviations.

### 3.4. Differential Scanning Calorimetry

Differential scanning calorimetry (DSC) measurements were carried out on a PerkinElmer differential scanning calorimeter DSC 8500 (PerkinElmer Inc., Waltham, MA, USA). The samples were cut from the shoulders of the injection-molded MPS and encapsulated in perforated aluminum pans. The average sample weight was around 8 mg. The procedure consisted of an initial heating phase, subsequent cooling, and a second heating phase, each in the temperature range of 0 °C to 200 °C with a constant heating/cooling rate of 10 K/min with nitrogen as the purge gas and a flow rate of 20 mL/min. The DSC measurements were accomplished to determine the melting peak in the second heating phase, which is characteristic of the semi-crystallinity achieved under controlled cooling in the DSC device. To determine the melting enthalpy of PP, first, the whole area of the melting peak was integrated with a temperature range from around 90 °C to 175 °C. Since all of the recyclates were contaminated with PE, the small PE peak was integrated from around 100 °C to 130 °C, which provides the PE melting enthalpy. The subtraction of the PE melting enthalpy from the above-mentioned whole area enthalpy provides the PP melting enthalpy. Due to the normalization of the heat flux via the specimen mass, the thermograms can be shown as normalized heat flux (W/g) over time (s), and the area of the peak (W/g·s) will calculate normalized melting enthalpy ΔH_m_ (J/g). For each material, five samples, each cut from an individual MPS, were used for the calculation of average values and standard deviations. The measurements were taken according to ISO 11357-1 [25] and ISO 11357-3 [26].

### 3.5. Oxidation Induction Temperature (Dynamic OIT) Measurements

The static OIT, or oxidation induction time, is mentioned within the pipe standards. For the static method, a sample is heated up to 200 °C under an inert nitrogen atmosphere and then, the isotherm at 200 °C, exposed to oxygen. All of the mentioned pipe standards demand that the pipe resin withstands at least 8 min within the oxygen atmosphere before it starts to oxidize, detected by an exothermal heat flux in, e.g., a DSC. While this method is well suited to show if a material is stabilized, the comparison of different materials can be tedious, as testing times strongly depend on the testing temperature; hence, testing at different temperatures is necessary to test different levels of stabilization. The dynamic OIT or oxidation induction temperature does not have this problem. Instead of measuring the time the sample needs for oxidation at a specific temperature, the sample is heated up in synthesized air or an oxygen environment until exothermal oxidation occurs. Instead of the onset time until oxidation, the onset temperature is compared. With this method, all levels of stabilization can be compared with the same test conditions.

While the dynamic method is not mentioned within the relevant pipe standards, it is well developed at the author’s institute and acknowledged in the literature [27,28] and is, therefore, used instead of the static method. For the experiments in this work, a differential thermal analysis (DTA) instrument from PerkinElmer, the DSC 4000, was utilized to characterize the oxidation induction temperature (dynamic OIT) according to ISO 11357-6 [9]. The samples were cut from the shoulders of the injection-molded MPS and encapsulated in perforated aluminum pans. The average sample weight was around 8 mg. A single heating step between 23 °C and 300 °C was performed with a heating rate of 10 K/min with synthetic air as purge gas and a flow rate of 20 mL/min. The point of intersection of the slope before oxidation and during oxidation provides the onset of oxidation or the oxidation induction temperature in °C. For each material, five samples, each cut from an individual MPS, were used for the calculation of the average values and standard deviations.

### 3.6. Tensile Tests

The tensile properties (tensile modulus, yield stress, and strain at break) were examined with a universal testing machine Zwick/Roell AllroundLine Z020, equipped with a Zwick/Roell multi-extensometer strain measurement system with MPS. The test parameters and MPS were used according to ISO 527-1 [14], ISO 527-2 [29], and ISO 19069 [22] with a traverse speed of 1 mm/min for tensile modulus determination until a strain of 0.25%, and after that 50 mm/min until failure. The calculations of tensile modulus, yield stress, and strain at break were undertaken in accordance with ISO 527-1. Therefore, the tensile modulus was calculated as the slope of the stress/strain curve between 0.05% and 0.25% via regression; the yield stress was the stress at the first occurrence of strain increase without a stress increase, and the strain at break was the strain when the specimen broke. The strain was recorded via a multi-extensometer until yield. From there, the nominal strain was calculated via Method B according to ISO 527-1 with the aid of the crosshead displacement. This process is integrated and automated in the used testing software TestXpert III (v1.61, ZwickRoell GmbH & Co. KG, Ulm, Germany). For each material, five MPSs were tested for the calculation of average values and standard deviations.

### 3.7. Charpy Impact Tests

Impact properties were determined according to ISO 179-1 [15] on a Zwick/Roell HIT25P pendulum impact tester. After pretests to determine the suitable pendulum size (absorbed energy between 10% and 80% of the available energy at impact), either a 2 Joule or a 5 Joule pendulum, the pendulum with the highest available energy that still conforms to these requirements, was chosen for testing the respective material. Notches were produced with a Leica RM2265 microtome (Leica Biosystems Nussloch GmbH, Nussloch, Germany) and measured with an Olympus SZX16 stereomicroscope (Olympus K.K., Tokyo, Japan). The test conditions were a 23 °C test temperature with the Type 1 specimen, edgewise blow direction, and notch Type A, i.e., a 0.25 mm notch radius or short ISO 179-1/1eA, which is one of the preferred methods of the standard. For each material, ten specimens were tested for the calculation of the average values and standard deviations.

### 3.8. Fatigue Crack Growth Experiments

For the investigation of the long-term mechanical property, the slow crack growth (SCG) resistance, fatigue crack growth (FCG) experiments, which measures the SCG resistance under cyclic loading, with cracked round bar (CRB) specimens following ISO 18489 [16], were conducted. The CRB specimens were tested with an electro-dynamic testing machine of the type Instron ElectroPuls E10000 (Illinois Tool Works Inc., Glenview, IL, USA). The ISO 18489 suggested load ranges are defined for polyethylene high-density (PE-HD) and would, if applied to the within this paper investigated PP compounds, lead to impractically long testing times. Therefore, the applicable load range for each material had to be determined by pretests. Eventually, sinusoidal loading profiles with a frequency of 10 Hz, an R-ratio of 0.1, and individually adjusted initial stress intensity factor ranges (ΔK_I_) were used to achieve testing times between 10 h and 100 h. For a better estimation of the applied force ranges: maximum forces of 2400 N to 1600 N were applied in order to reach these to other publications’ comparable testing times [30,31]. After the failure of the specimen, the actual initial crack length was measured with an Olympus SZX16 stereomicroscope, and ΔK_I_ was calculated afterward. The ΔK_I_ value depends on the geometry, applied force range, and initial crack length, as can be seen in the Formulas (2)–(4), developed by Benthem and Koiter [32] and used within the CRB test standard ISO 18489.

(2) $$\Delta K_{I}=\frac{\Delta F}{\pi \cdot b^{2}}\cdot \sqrt{\frac{\pi \cdot a_{\mathrm{ini}}\cdot b}{r}}\cdot f\left(\frac{b}{r}\right)$$

(3) $$b=r-a_{\mathrm{ini}}$$

(4) $$f\left(\frac{b}{r}\right)=\frac{1}{2}\cdot \left[1+\frac{1}{2}\cdot \left(\frac{b}{r}\right)+\frac{3}{8}\cdot {\left(\frac{b}{r}\right)}^{2}-0.363\cdot {\left(\frac{b}{r}\right)}^{3}+0.731\cdot {\left(\frac{b}{r}\right)}^{4}\right]$$

where ΔK_I_ is the initial stress intensity factor range in loading Mode I [33], ΔF the applied force range, a_ini_ the initial crack length, r is the radius of the specimen, b the ligament, and f(b/r) a geometry function.

Per material, at least three tests with different ΔK_I_, hence with different applied force ranges, were conducted to validate the method and to draw a linear approximation for comparison between the different materials. Characteristic double logarithmic FCG failure curves were plotted to provide the relationship between the initial stress intensity factor range, ΔK_I_ in MPa·m^0.5^ as a function of cycles to failure, N. The fracture surfaces were investigated with a Keyence VHX 7000 optical microscope (Keyence Corporation, Osaka, Japan).

## 4. Results

### 4.1. Introductory Results of the Recyclates

The recyclates were characterized before the formulation of the compounds. As these recyclates come from packaging applications, they show a distinctly different property profile than those shown in pipe compounds and virgin materials in the following chapters. Since their MFRs are much higher, specimen production is different and affects the resulting properties. Furthermore, a comparison of the pure recyclates with the pipe compounds leads to unfavorable diagram scales and, therefore, the properties of the three used recyclates are separately listed in Table 2.

### 4.2. Melt Flow Rate

The maximum tolerated MFR of the resin differs between the pipe standards. While the standard for in-house drainage [4] permits resins with an MFR of up to 3 g/10 min, the standards for underground drainage and sewerage pipes [5,6,7] only permit resins with an MFR of up to 1.5 g/10 min. The values should be measured according to ISO 1133-1 at 230 °C and with 2.16 kg. These values apply to resins used for both pipes and fittings.

**Figure 1:**
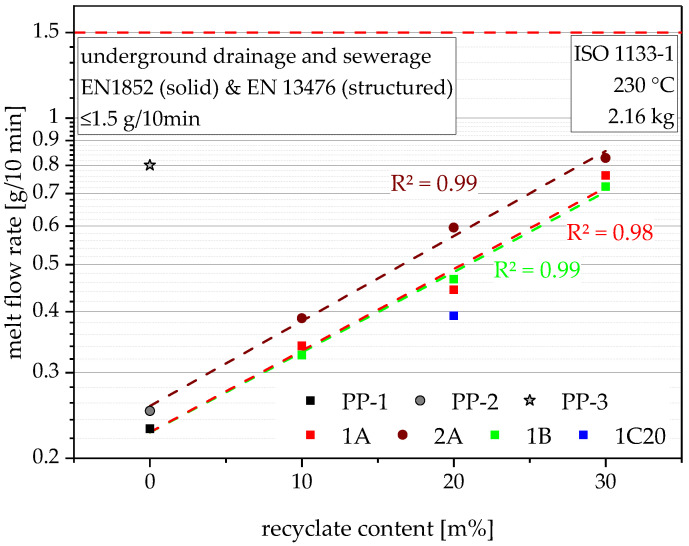
Graphical illustration of MFR values of the virgin blending partners PP-1 and PP-2, as well as the virgin injection molding grade PP-3 (as 0 m% recyclate content data points), and the compounds containing 10 m%, 20 m%, and 30 m% rPP-A, rPP-B, and rPO-C, respectively. Linear approximations of the compounding series 1A, 2A, and 1B are plotted as dashed lines, and the respective R^2^ values are shown in the matching colors.

**Figure 2:**
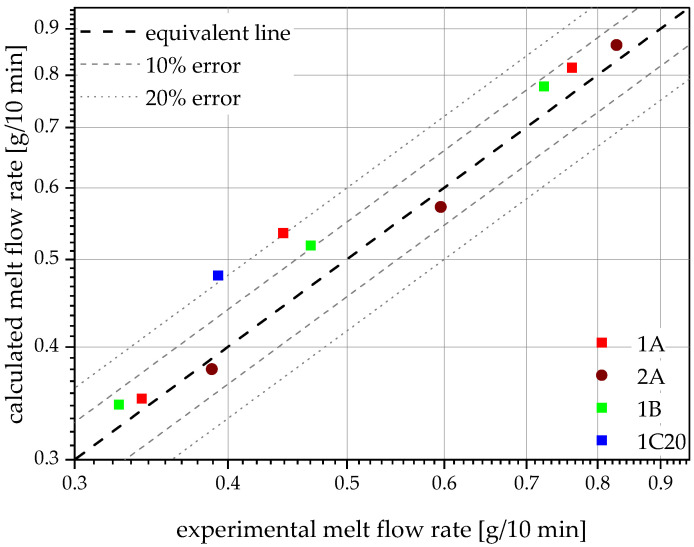
Plot of calculated (via Arrhenius mixing rule) vs. experimental MFR values. Most compounds lie within or around the 10% error range. Only 1A20 (0.54 g/10 min calculated vs. 0.44 g/10 min experimental MFR) and 1C20 (0.48 g/10 min calculated vs. 0.39 g/10 min measured MFR) show around 20% error.

Despite their mentioned data within the data sheets, all virgin materials and recyclates were measured together with the compounds for an accurate comparison. The used virgin PP pipe-grade PP-1 shows the lowest value with 0.23 g/10 min, followed by the second virgin PP pipe-grade PP-2 with 0.25 g/10 min, both shown at 0 m% recyclate content in Figure 1. The third PP pipe grade, which is used for injection molding of, e.g., fittings, was also tested and met the advertised MFR of 0.80 g/10 min. The pure recyclates are not shown in Figure 1, as their unproportionally high MFRs of 15.6 g/10 min (rPP-A), 13.3 g/10 min (rPP-B) and 9.1 g/10 min (rPO-C) would distort the graph and render the lower MFRs indistinguishable. The compounds show MFRs in the range of the Arrhenius mixing rule predicted values (see Figure 2) with rising MFRs for rising recyclate contents [34]. Therefore, linear fits within the logarithmic graph show high R^2^ values of 0.98 and 0.99. As predicted by the MFRs of the blending partners, compounds containing the lower MFR recyclates generally show lower MFRs at the same recyclate contents, except for 1B20, which shows a slightly higher MFR than 1A20. Furthermore, compounds made with PP-2 (shown as 2A) generally show higher MFRs at the same recyclate content than compounds made with PP-1 (shown as 1A). All of the produced compounds fulfill the lower MFR requirement from the discussed drainage and sewerage pipe standards, showing values well below 1.5 g/10 min.

### 4.3. Density

**Figure 3:**
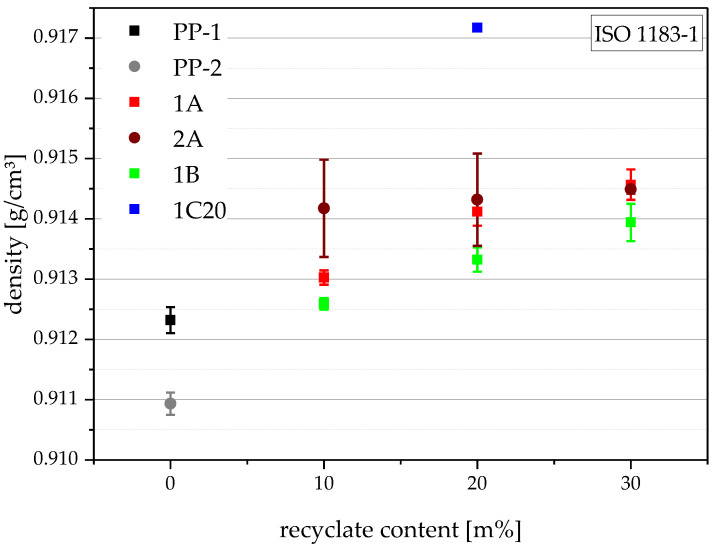
Graphical illustration of density values of the virgin blending partners PP-1 and PP-2 (as 0 m% recyclate content data points) and all compounds. The vertical bars show the sample standard deviations.

The density of polyolefins can be attributed to their crystallinity. Higher crystallinity means denser packing and, therefore, higher density [35]. Recyclates are often contaminated with organic (e.g., other, denser polymers, such as PE-HD, with ~0.96 g/mm^3^) and/or inorganic (e.g., carbon black and calcium carbonate) contaminants, which also raise the density [36]. The results of the density measurements, which are depicted in Figure 3, show a clear trend to higher densities with rising recyclate content.

PP-2 shows the lowest density of 0.9109 g/cm^3^, followed by PP-1 with 0.9123 g/cm^3^. This would suggest, baring fillers, that PP-1 has a higher crystallinity than PP-2. The compounding series that contain either rPP-A or rPP-B range from 0.9125 g/cm^3^ for 1B10 to 0.9146 g/cm^3^ for 1A30. The compounds containing the white rPP-B have lower densities at the same recyclate content than compounds containing rPP-A. If the compounding series 1A and 1B have similar crystallinities, this would suggest lower amounts of higher-density contaminants in rPP-B as in rPP-A. It seems that 2A10 has a higher density than 1A10, but the high standard deviation of the measurement discourages its veracity. The compound containing rPO-C, 1C20, shows by far the highest density of 0.9172 g/cm^3^, which would be explained by the alleged mixed feedstock containing PE.

### 4.4. Melting Behaviour

**Figure 4:**
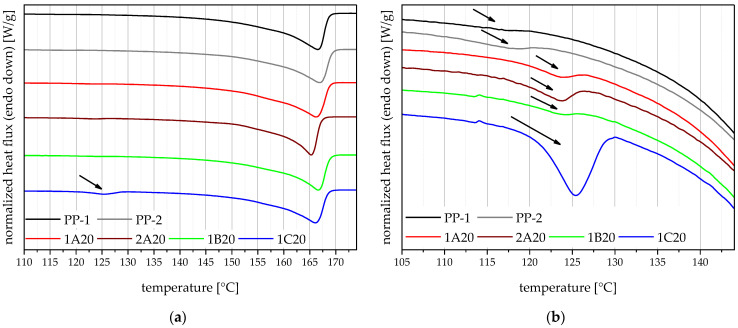
Thermograms of the virgin blending partners PP-1 and PP-2, and the four 20 m% recyclate compounds (**a**) and a more detailed look at the PE melting region of these thermograms (**b**). The arrows indicate PE melting peaks.

The DSC measurements are used to assess the crystallinity of the materials and detect foreign polymers. It is known that contaminations by other polymers can affect the performance of polyolefins [36]. All of the shown thermograms (the virgin mixing partner PP-1 and PP-2, as well as the four 20 m% recyclate compounds) are mostly dominated by a melting peak around 165 °C, identifying them as PP materials as seen in Figure 4a. In addition to that, 1C20 shows a pronounced endothermic peak at around 125 °C, indicating higher amounts of PE compared to the other compounds.

On a closer look, all the thermograms, even the virgin PP grades, exhibit quantifiable PE melting peaks, indicated by arrows within Figure 4b. This points out a high enough continuous ethylene segment content within the virgin PP block copolymers to form PE crystals. Furthermore, compared to the virgin PP grades, all compounds have larger PE melting peaks, indicating PE contaminations in all three recyclates.

**Figure 5:**
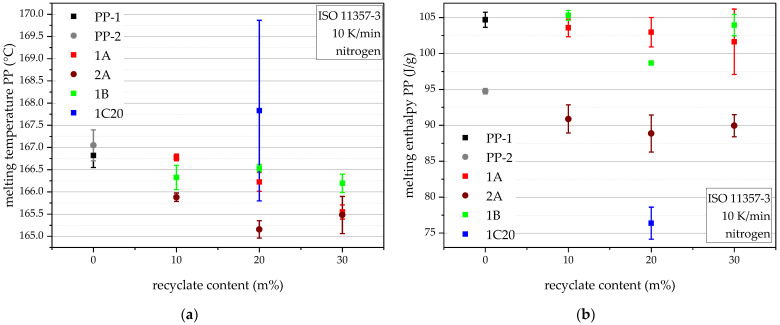
DSC results of the virgin blending partners and all compounds in terms of PP melting temperatures (**a**) and PP melting enthalpies (**b**). The vertical bars show the sample standard deviations.

The PP melting temperatures, while showing only small differences, follow the trend of lower PP melting temperature with rising recyclate contents, except for 1C20, as seen in Figure 5a. 1C20 seems to be more heterogeneous than the others, as the higher standard deviations suggest. The higher PP melting temperature would also suggest PP crystals with higher melting points, as in homo PP, and together with its high standard deviation, this is evidence for the diverse feedstock of rPO-C. The PP melting enthalpies, seen in Figure 5b, show the higher melting enthalpy of PP-1, compared to PP-2, which on the one hand, corroborates the density values (as higher melting enthalpy translates to higher crystallinity and, therefore, higher density [35]) and furthermore translates into the compounds made with the respective virgin blending partner (see 1A vs. 2A). 1C20 is again easily distinguishable by its significantly lower PP melting enthalpy.

**Figure 6:**
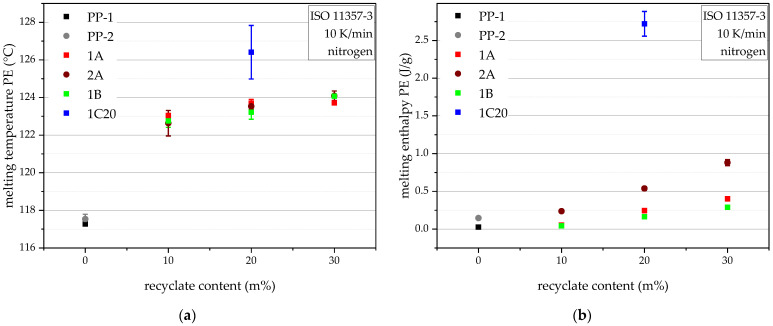
DSC results of the virgin blending partners and all compounds in terms of PE melting temperatures (**a**) and PE melting enthalpies (**b**). The vertical bars show the sample standard deviations.

The above for PP melting temperatures and enthalpies observed trends of lower temperature and enthalpy with rising recyclate content are reversed for PE melting temperatures and PE melting enthalpies and more pronounced, as seen in Figure 6a,b. The PE melting temperatures, as well as the PE melting enthalpies, rise with a rising recyclate content. This suggests a rising level of PE contamination with rising recyclate content. 1A shows higher PE melting enthalpies than 1B and 2A significantly more than 1A. These differences become even more pronounced with higher recyclate content in the compound. Furthermore, 1C20 shows both the highest PE melting temperature and enthalpy, once more an affirmation of its mixed waste feedstock and in agreement with the measured density values.

### 4.5. Oxidation Induction Temperature (Dynamic OIT)

**Figure 7:**
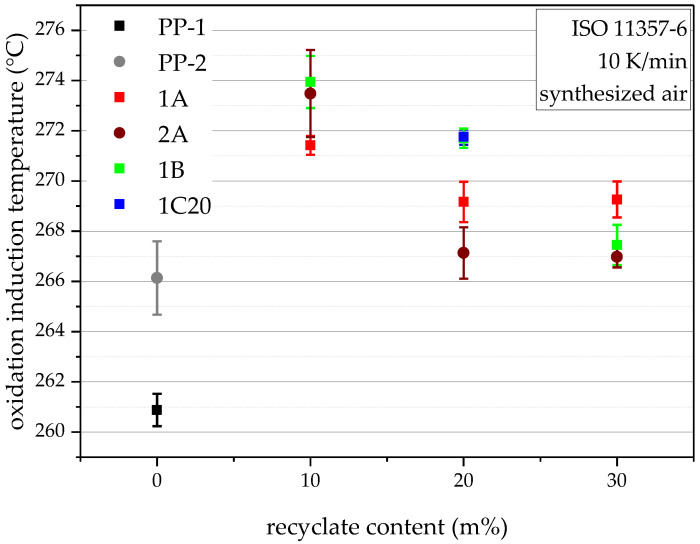
Graphical illustration of oxidation induction temperatures of the virgin blending partners PP-1 and PP-2 (as 0 m% recyclate content data points), and all compounds. The vertical bars show the sample standard deviations.

The results of the oxidation induction temperature experiments, shown in Figure 7, primarily depict the effectiveness of the added and inherent stabilizers. Unlike the results shown before, during compounding, the added stabilizers, in combination with the high virgin content, leads to better than virgin results for all compounds. PP-1 has the lowest dynamic OIT with 260.9 °C, followed by PP-2 with 266.1 °C. The more recyclate in the compounds, the lower the dynamic OIT. Therefore, the lowest dynamic OITs within the compounds are shown by the 30 m% compounds with ~267 °C, followed by the 20 m% compounds with dynamic OITs between 267.1 °C and 271.8 °C, and finally, the highest dynamic OITs are shown by the 10 m% compounds with up to 273.9 °C. These results verify the stability against oxidation of all produced compounds.

### 4.6. Tensile Properties

**Figure 8:**
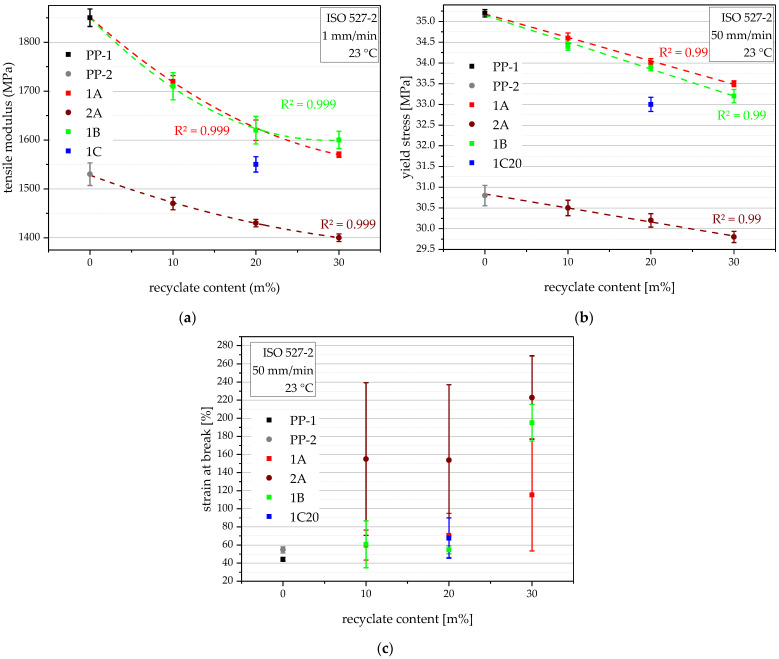
Graphical illustration of tensile modulus (**a**), yield stress (**b**), and strain at break values (**c**) of the virgin blending partners PP-1 and PP-2 (as 0 m% recyclate content data points) and all compounds. The vertical bars show the sample standard deviations. Polynomial (**a**) and linear (**b**) approximations depicting the three distinguished compounding series (1A, 2A, 1B) are plotted as dashed lines, and the respective R^2^ values are shown in matching colors.

The tensile properties of the compounds are very important, as the ring stiffness is derived from it, and the other failure parameters depend on the yield stress. The virgin pipe-grade benchmark PP-1 excels with its high tensile modulus of 1850 MPa, while the second benchmark, PP-2, shows a much lower value of only 1530 MPa (17% lower), as seen in Figure 8a. The compounds inherit the high tensile modulus from their virgin blending partners and show lower tensile moduli with rising recyclate content. Therefore, the tensile moduli of the compounding series 1A and 1B range from 1720 MPa at 10 m% recyclate content to 1570 MPa at 30 m% recyclate content. 1C20 is the exception, with an even lower 1550 MPa, especially compared to the other compounds at 20 m%, which could be explained with its higher PE content. The compounds produced with PP-2, so the compounding series 2A, show the lowest tensile moduli ranging from 1470 MPa at 10 m% recyclate content to 1400 MPa at 30 m% recyclate content. The decrease in stiffness shows for the compounding series 1A, 2A, and 1B a polynomial trend of the second order with a high R^2^ for each of them. The different slopes between 1A and 2A can be explained by the varying differences in tensile modulus between the respective blending partners.

The same trends, although linear, also have a high R^2^ of 0.99 for each of them, show for the yield stresses, where PP-1 excels with 35.2 MPa, while PP-2 only shows 30.8 MPa (13% lower), as seen in Figure 8b. The same can also be said for the compounds. The yield stresses of the compounding series 1A and 1B range from 34.6 MPa at 10 m% recyclate content to 33.2 MPa at 30 m% recyclate content. 1C20 is again the exception, with an even lower 33.0 MPa, which could again be explained by its higher PE content. The compounding series 2A shows the lowest values ranging from 30.5 MPa for 10 m% recyclate content to 29.8 MPa at 30 m% recyclate content.

The strain at break values, as seen in Figure 8c, show different trends. As the resins MFR affects the flow into the specimen mold and hence the orientation, lower MFR resins tend to show lower strain at break values [18]. Furthermore, impurities can lead to the premature failure of the specimen, and their random occurrence in specimens leads to high standard deviations, as seen in compounding series 2A.

### 4.7. Charpy Notched Impact Strength

**Figure 9:**
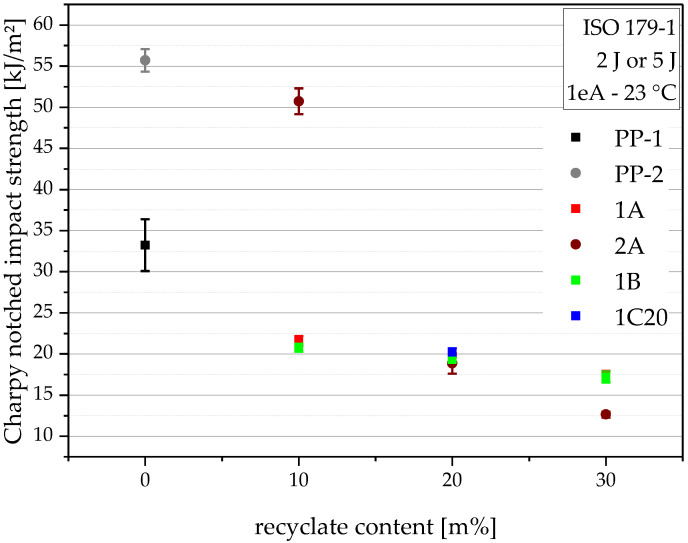
Graphical illustration of Charpy notched impact strength of the virgin blending partners PP-1 and PP-2 (as 0 m% recyclate content data points) and all compounds. The vertical bars show the sample standard deviations.

The Charpy notched impact strength of the virgin blending partners and all of the compounds are depicted in Figure 9. The results show the main asset of PP-2, its very high impact strength of 55.7 kJ/m^2^. The other virgin, PP-1, only delivers 33.2 kJ/m^2^ (40% lower). This difference is also the main reason why these two blending partners were acquired to test separate compounding series with the same recyclate. All of the compounds that incorporated PP-1 show decreasing impact performance with increasing recyclate content, from 21.6 kJ/m^2^ for 10 m% recyclate content to 17.2 kJ/m^2^ at 30 m% recyclate content. 2A10, the compound containing PP-2 and 10 m% recyclate, also shows a very high impact strength of 50.7 kJ/m^2^ and therefore complies with the high expectations given from using 90 m% of the impact-resistant PP-2. However, the 2A compounds containing 20 m% and 30 m% recyclate content show similarly low (18.8 kJ/m2 for 2A20) and even lower (12.6 kJ/m^2^ for 2A30) impact strengths than the compounds with PP-1. Hence, it is obvious that compounds with PP-2 are failing to translate the initially high impact strength to compounds with higher recyclate content.

### 4.8. Fatigue Crack Growth Resistance

**Figure 10:**
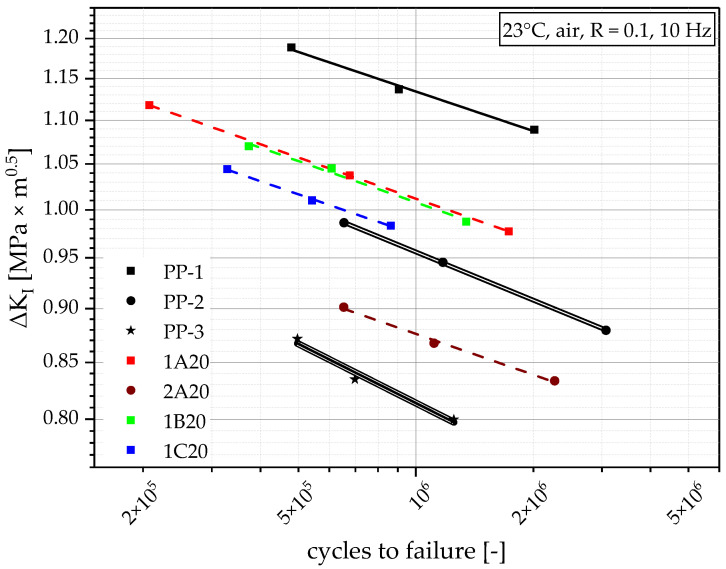
Graphical illustration of FCG resistance of the virgin blending partners PP-1 and PP-2, as well as the injection molding benchmark PP-3 and all compounds with 20 m% recyclate content. Initially applied ΔK_I_ as a function of cycles to failure. All lines are linear approximations between three measurement points.

FCG experiments on CRB specimens from the virgin materials show a broad range of FCG resistances and, for a first comparison, can be seen together with the four compounds with 20 m% recyclate content in Figure 10. The points within Figure 10 show the individual result (cycles to failure at the tested ΔK_I_) from each measurement, and the lines show the linear approximation between the corresponding individual results (at least three). The results from Figure 10 show the big difference between the virgin grades and where the 20 m% compounds can be found within that range. Here, it is clearly distinguishable that PP-1 has the highest FCG resistance (highest loading for comparable cycles to failure), followed by the two similarly performing compounds, 1A20 and 1B20. 1C20 still shows a little bit higher FCG resistance than PP-2, and the PP-2 compound 2A20 still delivers a higher FCG resistance than the lowest-ranking PP-3.

**Figure 11:**
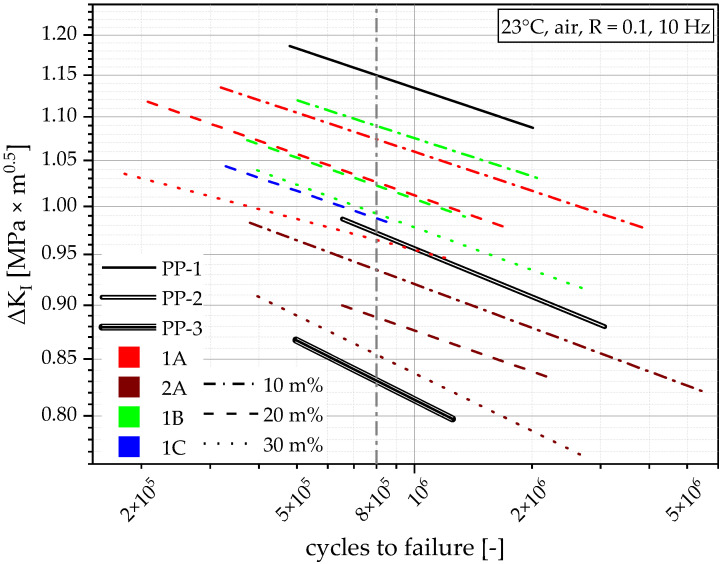
Graphical illustration of the FCG resistance of all tested materials within this work. Initially applied ΔK_I_ as a function of cycles to failure. All lines, except the vertical grey line, are linear approximations between at least three measurement points. The vertical grey line shows 8 × 10^5^ cycles.

Figure 11 shows the same graph with data from all the compounds. To make the graph clearly arranged, the data points are hidden, and only the linear approximations are shown. Within every compounding series, the material with the lower recyclate content performs best. The performance of compounding series 2A has the worst material performance of all compounds. Nevertheless, it appears to have a higher FCG resistance than the virgin benchmark PP-3. The other compounds are harder to differentiate. To achieve comparability while maintaining economical testing times, a greater variation in the loading must be applied, which results in a graph with vertically aligned data, as seen in Figure 11. Since a comparison of all materials at the same ΔK_I_ is not possible due to the vertical spread of the data, they will be compared at the same cycles (8 × 10^5^ cycles), as indicated by the grey dot-dashed line in Figure 11. PP-1 shows by far the highest ΔK_I_ at 8 × 10^5^ cycles with 1.15 MPa × m^0.5^. The other virgin polymers show far lower values, with 0.97 MPa × m^0.5^ for PP-2 (15% lower) and 0.83 MPa × m^0.5^ for PP-3 (28% lower). The results of 1A, 1B, and 1C show ΔK_I_ at 8 × 10^5^ cycles from as high as 1.09 MPa × m^0.5^ (1B10, 7% lower than PP-1) to as low as 0.96 MPa × m^0.5^ (1A30, 16% lower than PP-1). The compounds produced with PP-2 show much lower values between 0.93 MPa × m^0.5^ (2A10, 19% lower than PP-1) and 0.85 MPa × m^0.5^ (2A30, 26% lower than PP-1), meaning that they are worse than PP-2 but better than PP-3. More informative ways to quantify and compare the FCG resistance are shown in the Discussion.

**Figure 12:**
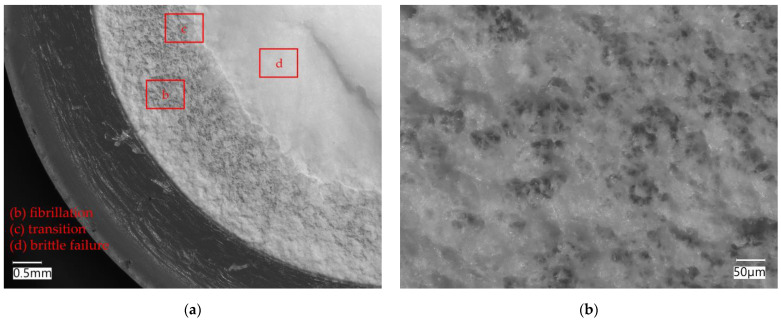

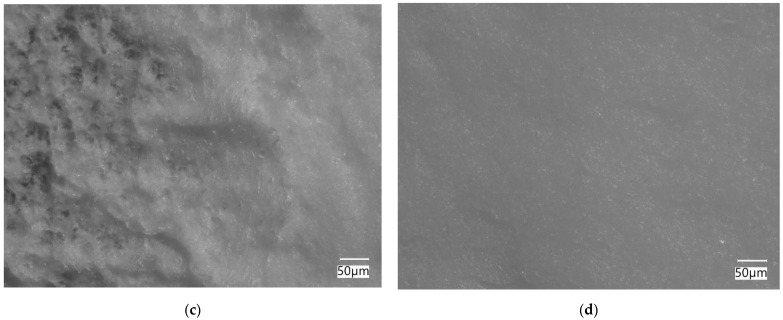
Images depicting the different crack growth mechanisms in a PP-1 specimen at ΔK_I_ of 1.09 MPa × m^0.5^. Overview image of specimen (**a**) the approximate positions of the three areas from fibrillation (**b**), transition (**c**), and large-scale brittle failure (**d**) are depicted with red rectangles.

The fracture surfaces can be roughly divided into three areas, as seen in Figure 12. Figure 12a shows a quarter of a specimen where the different areas are already discernable by red rectangles. The area close to the notch is dominated by fibrillation, as can be seen in Figure 12b. The fibrillation grows rougher and deeper, shown by the shadowed spots, the closer it is to the center of the specimen and as the crack growth progresses. Figure 12c shows the transition from the area of slow crack growth indicated by fibrillation to the area of abrupt failure at the end of the test, which is also depicted in Figure 12d. This third area shows a smooth surface which indicates a large-scale brittle failure. These three areas can also be observed in other published literature concerning CRB experiments on PP [30,31].

## 5. Discussion

**Figure 13:**
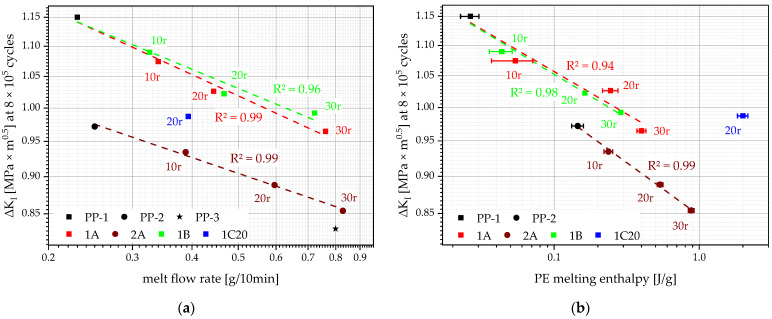
Correlations of ΔK_I_ at 8 × 10^5^ cycles with the respective MFR (**a**), as well as with the PE melting enthalpy (**b**) of the shown materials. The horizontal bars show the sample standard deviations. Linear approximations of the compounding series 1A, 2A, and 1B are plotted as dashed lines, and the respective R^2^ values are shown in the matching colors.

A way of quantifying FCG resistance is to compare the linear approximations at the same initial loading (ΔK_I_) or at the same number of cycles. In our case, FCG data will be compared at the same number of cycles (8 × 10^5^ cycles), as indicated by the grey dash-dotted line in Figure 11. The thereafter gathered data from the different materials ΔK_I_ at 8 × 10^5^ cycles can be correlated with other values inherent to the respective material, e.g., the MFR, as seen in Figure 13a, or the PE melting enthalpy as seen in Figure 13b.

The dashed lines in Figure 13a show the linear approximations from 0 m% to 30 m% recyclate content. While the compounding series 1A, 2A, and 1B show excellent trends of increasing MFR with decreasing ΔK_I_ at 8 × 10^5^ cycles, it does not explain why 1C20 and the whole compounding series of 2A have worse performances than 1A and 1B. Even at lower MFR values (1C20 and 2A10), these compounds endure a lower ΔK_I_ for 8 × 10^5^ cycles than, e.g., 1A20, 1B20, and even 1B30.

The other correlation between ΔK_I_ at 8 × 10^5^ cycles and PE melting enthalpy, as shown in Figure 13b, explains these discrepancies. The compounding series 1A, 2A, and 1B show trends of increasing PE melting enthalpy with decreasing ΔK_I_ at 8 × 10^5^ cycles. The position of 1C20 in this graph suggests that its performance (ΔK_I_ at 8 × 10^5^ cycles) is too good for its given PE melting enthalpy. The combination of its relatively low MFR (compared to other PP-1 compounds at this performance) and its relatively high PE melting enthalpy (compared to other PP-1 compounds at this performance) balance each other out and explain the altogether mediocre performance of 1C20. The performance, according to both MFR and PE melting enthalpy, of the underperforming compounding series 2A cannot be explained with these two correlations alone. It is assumed that other mechanical parameters, such as tensile modulus and yield stress, which are both much higher for PP-1 compounds than for PP-2 compounds, make up for this difference.

## 6. Conclusions

Polypropylene (PP) recyclates from packaging waste streams usually have high melt flow rates (MFR) and low resistances against fatigue crack growth (FCG), both properties that are relevant for pipe applications. The blending of these recyclates with low-MFR, FCG-resistant virgin pipe-grade PP creates compounds that show good FCG resistance. Although the FCG resistances of virgin pipe grades are highly optimized and dependent on chemical and morphological factors [37], the performance of the compounds with rPP tested in this paper is mostly described by its MFR and the PE melting enthalpy, thus cross-contamination. However, other influences, such as the tensile modulus and yield stress, can also attribute to the performance differences within the compounds. This is a highly complex topic depending a lot on the quality of the used recyclate, contaminations, fillers, etc. Therefore, this is an intermediate step in the development and investigations regarding the use of PCRs in non-pressure applications. Especially the batch-to-batch variation of the used PCRs needs to be considered regarding risk evaluation and displayed accordingly in an assessment of the conformity testing of PCRs and recyclate compounds.

All of the produced compounds were able to achieve a higher FCG resistance than the virgin injection molding pipe-grade PP-3. Furthermore, all of the compounds produced with virgin pipe-grade PP-1 were also able to achieve higher FCG resistances than virgin pipe-grade PP-2. These FCG resistance results, together with the short-term mechanical properties and the additional investigated property profile, show that some compounds can be considered for pipe applications. Nevertheless, the authors want to state that while the here presented results of the produced compounds seem to fulfill the requirements to be used as pipe-grade material, many other properties, in particular tests conducted on produced pipes, are necessary for successful pipe production and admission as pipe resin. Furthermore, for any given recyclate grade, its consistency remains to be investigated to ensure the stability of the pipe quality.

## Figures and Tables

**Table 1:** List of compounds and their respective amounts of blending partners. To all compounds, additional 0.15 m% Irganox^®^ 1010 long-term stabilizer [20] and 0.15 m% Irgafos^®^ 168 processing stabilizer [21] were added during compounding.

	PP-1	PP-2	rPP-A	rPP-B	rPO-C
	m%	m%	m%	m%	m%
1A10	90	-	10	-	-
1A20	80	-	20	-	-
1A30	70	-	30	-	-
1B10	90	-	-	10	-
1B20	80	-	-	20	-
1B30	70	-	-	30	-
1C20	80	-	-	-	20
2A10	-	90	10	-	-
2A20	-	80	20	-	-
2A30	-	70	30	-	-

PP-1 and PP-2 are virgin drainage pipe grades. rPP-A and rPP-B are recyclates derived from PP packaging waste. rPO-C is a recyclate derived from mixed PE and PP packaging waste.

**Table 2:** Properties of the used recyclates.

	rPP-A	rPP-B	rPO-C
melt flow rate [g/10 min]	15.8 ± 0.1	13.3 ± 0.3	9.1 ± 0.1
tensile modulus [MPa]	1170 ± 13	1320 ± 45	1010 ± 0
yield stress [MPa]	27.2 ± 0.1	28.1 ± 0.3	22.3 ± 0.1
strain at break [%]	46.5 ± 15.2	38.9 ± 34.4	13.7 ± 0.5
Charpy notched impact strength [MPa]	6.2 ± 0.2	6.8 ± 0.3	5.5 ± 0.4
oxidation induction temperature [°C]	209.5 ± 2.5	211.6 ± 0.2	210.8 ± 0.5

rPP-A and rPP-B are recyclates derived from PP packaging waste. rPO-C is a recyclate derived from mixed PE and PP packaging waste.

## Data Availability

No additional data available.
